# Compositional analysis of genetic ancestry shows that relative proportion of African ancestry is positively associated with fecal myeloperoxidase in Brazilian infants

**DOI:** 10.64898/2026.10.02.26364607

**Published:** 2026-10-05

**Authors:** Josyf C. Mychaleckyj, Alexandre Havt, Uma Nayak, Richard L. Guerrant, Aldo A. M. Lima

## Abstract

Genetic ancestry composition has been used extensively as a predictor of clinical outcomes, multiomic biomarkers, and endophenotypes, but the models have most often used untransformed ancestry fractions as explanatory variables. Under a simplex constraint results from this type of model can be uninterpretable and misleading. We demonstrate the use of multiple transformed log ratio coordinates of ancestral proportions as predictors in a study of excreted myeloperoxidase (MPO), a neutrophilic enzyme used as a fecal biomarker to measure intestinal inflammation and enteropathy. We show how to convert these difficult-to-interpret effects into association results for shifts between two ancestries under an isoancestry constraint (holding an ancestry fraction constant) using post-estimation average marginal effects and explain their geometric interpretation. We found that increasing proportion of African ancestry was associated with increased levels of excreted MPO in low-income household infants in three Northeastern Brazil studies, with a smaller negative secondary association of European ancestry, all under a tri-continental ancestry composition (African, European, Amerindian). While the maximum log(MPO) outcome variance explained by differences in genetic ancestry was modest at a few percent, we show evidence that these differences in heritability were context-dependent on measurement of MPO under active enteric infection. The results were attenuated by socio-economic status, but a signal remained. Overall, we found that African genomes carry additional alleles that enable a more intensive neutrophil response to enteric infection relative to European and Amerindian genomes, illuminating a possible host genetic background role in modulating immune response to enteric diseases and pathogens.

## Full Text Availability

The license terms selected by the author(s) for this preprint version do not permit archiving in PMC. The full text is available from the preprint server.

